# Cryptic species diversity in the *Hypsolebiasmagnificus* complex, a clade of endangered seasonal killifishes from the São Francisco River basin, Brazilian Caatinga (Cyprinodontiformes, Aplocheilidae)

**DOI:** 10.3897/zookeys.777.25058

**Published:** 2018-07-30

**Authors:** Wilson J. E. M. Costa, Pedro F. Amorim, José Leonardo O. Mattos

**Affiliations:** 1 Laboratory of Systematics and Evolution of Teleost Fishes, Institute of Biology, Federal University of Rio de Janeiro, Caixa Postal 68049, CEP 21941-971, Rio de Janeiro, Brazil Federal University of Rio de Janeiro Rio de Janeiro Brazil

**Keywords:** Biodiversity, conservation, molecular taxonomy, species delimitation, systematics

## Abstract

A great diversity of animal species adapted to life in the semi-arid Caatinga of northeastern Brazil, including seasonal killifishes, has been reported in the last three decades. More recently, field and molecular data have shown a high occurrence of cryptic species. The killifish group herein analysed, the *Hypsolebiasmagnificus* species complex, is endemic to the middle and southern portion of the Caatinga, occupying about 120 km along the floodplains of the middle São Francisco River and some adjacent tributaries. Species of this complex are rare and presently considered threatened with extinction, being uniquely found in pools protected by trees and bushes. Single-locus delimitation methods were used to test species limits of populations displaying different colour patterns along the whole distribution of the complex. All analyses consistently supported the three nominal species and two new, herein described: *H.gardneri* Costa, **sp. n.**, from the floodplains of the middle São Francisco River and *H.hamadryades* Costa, **sp. n.**, from the Gorotuba River floodplains. The phylogenetic analysis highly supports *H.hamadryades* as sister to a clade comprising *H.gardneri* and *H.harmonicus*. Our field observations suggest that *H.hamadryades* is a miniature species. This study indicates that the *H.magnificus* complex comprises cryptic species apparently endemic to small areas and extremely vulnerable to environmental changes, deserving high concern.

## Introduction

Recent studies on the fauna of the Caatinga, a biogeographical province of northeastern Brazil, have revealed a high diversity of species adapted to survive in semi-arid conditions, including small terrestrial vertebrates (Rodrigues 2003; Nascimento et al. 2013; Werneck et al. 2015) and seasonal killifishes (Costa 2001; Rosa et al. 2003; Costa et al. 2012, 2018). Seasonal killifishes live in temporary pools formed at rainy seasons, where they complete their entire life cycle, surviving as resistant eggs during dry periods (Wourms 1972; Costa 1995). In the Caatinga, pools disappear during a long dry season, frequently between May and October in most of the southern and central part of the Caatinga, which concentrates the great diversity of seasonal killifish species, but a short dry period may also occur between January end and February, with most pools drying again (Costa 2017).

*Hypsolebias* Costa, 2006 is the most species rich and morphologically diverse seasonal killifish genus in the Caatinga, where it is represented by two clades highly supported by morphological and molecular data (Costa 2006; Costa et al. 2017), the *H.antenori* group (Costa 2007; Costa et al. 2014, 2018) and the *H.magnificus* group (Costa 2007). Among species of this clade is *H.magnificus* (Costa & Brasil, 1991) and two closely related species, *H.picturatus* (Costa, 2000) and *H.harmonicus* (Costa, 2010) (Costa 2007, 2010), which together form a species complex, herein called the *H.magnificus* complex (hereafter HMC), distinguished from all other congeners by the presence of a narrow black margin on unpaired fins and pectoral fin in males (vs. black margin absent) and unpaired fins rounded or slightly pointed (vs. sharply pointed) (Costa 2010). Like other closely related congeners, HMC species live only in shadowed parts of the pools, protected by dense concentration of shrubs and trees (pers. obs. 1994–2018).

The first species of HMC to be described was *H.magnificus*, collected in the São Francisco River floodplains near the village of Mocambinho (Costa and Brasil 1991). With the discovery of *H.picturatus* and more frequent field studies between 1999 and 2005, the distribution of the complex was amplified to include records about 420 km N from the type locality of *H.magnificus* (Costa, 2007). During field studies between 2005 and 2010, a strong decline of natural habitats was progressively recorded, resulting in extinction of several populations of seasonal killifishes (Costa et al. 2012; Costa 2017). During this same period, a third species, *H.harmonicus*, was recognised and described (Costa 2010). However, the identity of two populations remained dubious. The first one, sharing a similar colour pattern with *H.harmonicus*, was collected in 2010 only about 40 km from the type locality of this species. The second one was tentatively identified as *H.magnificus* (Costa, 2017), but collected about 120 km from its type locality, at the upper Rio Gorotuba floodplains in 2017. However, fish from both populations exhibited a few distinct morphological characters suggesting that they are cryptic species (sense Bickford et al. 2006). Herein we analyse a segment of the mitochondrial gene cytochrome b for representatives of all nominal species of HMC and the two putative cryptic species, under different single-locus methods of species delineation in order of to provide a more accurate picture about species diversity in the complex.

## Material and methods

Specimens were captured with small dip nets (40 × 30 cm) and were euthanized soon after collection, using a buffered solution of tricaine methanesulfonate (MS-222) at a concentration of 250 mg/l, for a period of about 10 minutes (i.e. until opercular movements ceased). Representative live specimens were kept alive for about 24 hours, photographed, and then euthanized as described above. Specimens were fixed in 10 % formalin for a period of 10 days, and then transferred to 70 % ethanol, except for those used in the molecular analysis, fixed and preserved in 98 % ethanol. Collections were made with permits provided by ICMBio (Instituto Chico Mendes de Conservação da Biodiversidade; permit numbers: 34270-4, 20618-1, 57099-1) and methods for euthanasia were approved by CEUA-CCS-UFRJ (Ethics Committee for Animal Use of Federal University of Rio de Janeiro; permit number: 01200.001568/2013-87). Material is deposited in the ichthyological collections of: Instituto de Biologia, Universidade Federal do Rio de Janeiro, Rio de Janeiro (UFRJ) and Centro de Ciências Agrárias e Ambientais, Universidade Federal do Maranhão, Chapadinha (CICCAA). In lists of material, the abbreviation C&S indicates specimens prepared for osteological analysis and preserved in glycerine (see below), and DNA indicates specimens fixed and preserved in 98% ethanol. List of specimens used in the molecular analysis and their respective GenBank accession numbers appears in Table 1.

**Table 1.** List of specimens used in the molecular analysis, with their respective catalogue numbers, coordinates of the collecting site, and GenBank accession numbers for cytb sequences.

Species	Catalogue number	Coordinates	Cytb
* Hypsolebias carlettoi *	UFRJ 6780.2	14°13'42"S, 42°55'12"W	MH048856
* Hypsolebias fulminantis *	UFRJ 6726.1	14°12'21"S, 42°45'42"W	MH048854
* Hypsolebias gardneri *	UFRJ 6796.1	14°17'39"S, 43°42'32"W	MH048861
* Hypsolebias gardneri *	UFRJ 6796.3	14°17'39"S, 43°42'32"W	MH048862
* Hypsolebias gardneri *	UFRJ 6796.4	14°17'39"S, 43°42'32"W	MH048863
* Hypsolebias hamadryades *	UFRJ 11473.1	15°48'06"S, 43°19'19"W	MH048860
* Hypsolebias hamadryades *	UFRJ 11473.2	15°48'06"S, 43°19'19"W	MH048859
* Hypsolebias hamadryades *	UFRJ 11473.3	15°48'06"S, 43°19'19"W	MH048858
* Hypsolebias hamadryades *	UFRJ 11473.4	15°48'06"S, 43°19'19"W	MH048857
* Hypsolebias harmonicus *	UFRJ 6705.3	13°15'42"S, 43°31'00"W	MH048864
* Hypsolebias harmonicus *	UFRJ 6705.4	13°15'42"S, 43°31'00"W	MH048865
* Hypsolebias hellneri *	UFRJ 6700.2	15°04'50"S, 44°04'40"W	MH048855
* Hypsolebias magnificus *	UFRJ 6712.1	14°55'20"S, 43°29'56"W	MH048866
* Hypsolebias magnificus *	UFRJ 6712.2	14°55'20"S, 43°29'56"W	MH048867
* Hypsolebias picturatus *	UFRJ 6708.1	11°28'03"S, 43°17'10"W	MH048868

Descriptions of colouration in living fish were based on photographs of both sides of individuals. Photographs of at least two males and two females were taken in small aquaria about 24 hours after collection. Additional direct observations were made with fish in small transparent plastic bottles just after collection. Measurements and counts follow Costa (1988). Measurements are presented as percentages of standard length (SL), except for those related to head morphology, which are expressed as percentages of head length. Measurements were made only in well preserved adult specimens; juvenile specimens (less than 20 mm SL) and specimens presenting deformities were not measured. Fin-ray counts include all elements. At least four specimens, two males and two males, were cleared and stained for osteological analysis using Taylor and Van Dyke’s (1985) protocol. Terminology for osteological structures followed Costa (2006), for frontal squamation Hoedeman (1958), and for cephalic neuromast series Costa (2001). Osteological characters used in species descriptions are those that show informative variability in *Hypsolebias* (e.g., Costa 2006).

Total genomic DNA was extracted from muscle tissue of the right side of the caudal peduncle using the DNeasy Blood & Tissue Kit (Qiagen) according to the manufacturer instructions. To amplify a fragment of the mitochondrial DNA gene cytochrome b (cytb), we used the primers L14724 and H15149 (Kocher et al. 1989; Meyer et al. 1990). Polymerase chain reaction (PCR) was performed in 15 μl reaction mixtures containing 5× Green GoTaq Reaction Buffer (Promega), 3.2 mM MgCl_2_, 1 μM of each primer, 75 ng of total genomic DNA, 0.2 mM of each dNTP and 1 U of Taq polymerase. The thermocycling profile was: (1) 1 cycle of 4 minutes at 94 °C; (2) 35 cycles of 1 minute at 92 °C, 1 minute at 44–54 °C and 1 minute at 72 °C; and (3) 1 cycle of 4 minutes at 72 °C. In all PCR reactions, negative controls without DNA were used to check for contaminations. Amplified PCR products were purified using the Wizard SV Gel and PCR Clean-Up System (Promega). Sequencing reactions were made using the BigDye Terminator Cycle Sequencing Mix (Applied Biosystems). Cycle sequencing reactions were performed in 10 μl reaction volumes containing 1 μl BigDye 2.5, 1.55 μl 5× sequencing buffer (Applied Biosystems), 2 μl of the amplified products (10–40ng), and 2 μl primer. The thermocycling profile was: (1) 35 cycles of 10 seconds at 96 °C, 5 seconds at 54 °C and 4 minutes at 60 °C. The sequencing reactions were purified and denatured and the samples were run on an ABI 3130 Genetic Analyzer. Sequences were edited using MEGA 6 (Tamura et al. 2013) and aligned using ClustalW (Chenna et al. 2003); alignments were subsequently translated into amino acids residues to check premature stop codons or indels. List of specimens used in the molecular analysis and their respective GenBank accession numbers appear in Table 1.

Analyses were performed with a cytb fragment (463 bp), which has been efficiently used for delimitating cryptic species of different aplocheiloid killifish groups (Sonnenberg et al. 2006; Sonnenberg 2007; Van der Zee and Sonnenberg 2011; Costa et al. 2012, 2014; Agnèse et al. 2013). Terminal taxa were 12 specimens of five populations representing all nominal species of the HMC; out-groups comprised *H.carlettoi* (Costa & Nielsen, 2004) and *H.fulminantis* (Costa & Brasil, 1993), two species closely related to HMC (Costa 2006; Costa et al. 2017), and *H.hellneri* (Berkekamp, 1993), which was sister to all other members of the *H.magnificus* group (Costa 2006; Costa et al. 2017), was used to root the phylogeny. The best-fit model of sequence evolution was calculated by jModelTest 2.1.7 (Darriba et al. 2012), which indicated the general-time reversible model with a gamma frequency distribution of categories among sites (GTR + G). We inferred tree topology using Bayesian reconstruction performed with BEAST v.1.8 (Drummond et al. 2012), using an uncorrelated relaxed lognormal model and other parameters set as default; the MCMC length was 30,000,000 runs with sampling every 1000 runs. The quality of the MCMC chains was evaluated in Tracer 1.5 (Rambaut et al. 2013); a 25% burn-in was removed and the final tree was obtained using TreeAnnotator v.1.5 from BEAST v.1.8 package; support values of the Bayesian inference (BI) analysis were calculated by posterior probability. The following single-locus models for species delimitation were used: the Generalized Mixed Yule-Coalescent (GMYC) (Fujisawa and Barraclough 2013), independently applying both single and multiple-threshold, and the Bayesian implementation of Poisson Tree Process (bPTP) (Zhang et al. 2013), with 500,000 Markov chain Monte Carlo (MCMC) generations, thinning set to 100 and a burn-in of 25% initial samples, checking both Maximum likelihood and Bayesian solutions. All analyses were carried on the Exelixis Lab’s web server (GMYC at http://species.h-its.org/gmyc/; bPTP at http://species.h-its.org/ptp/).

## Results

**Figure 1.**
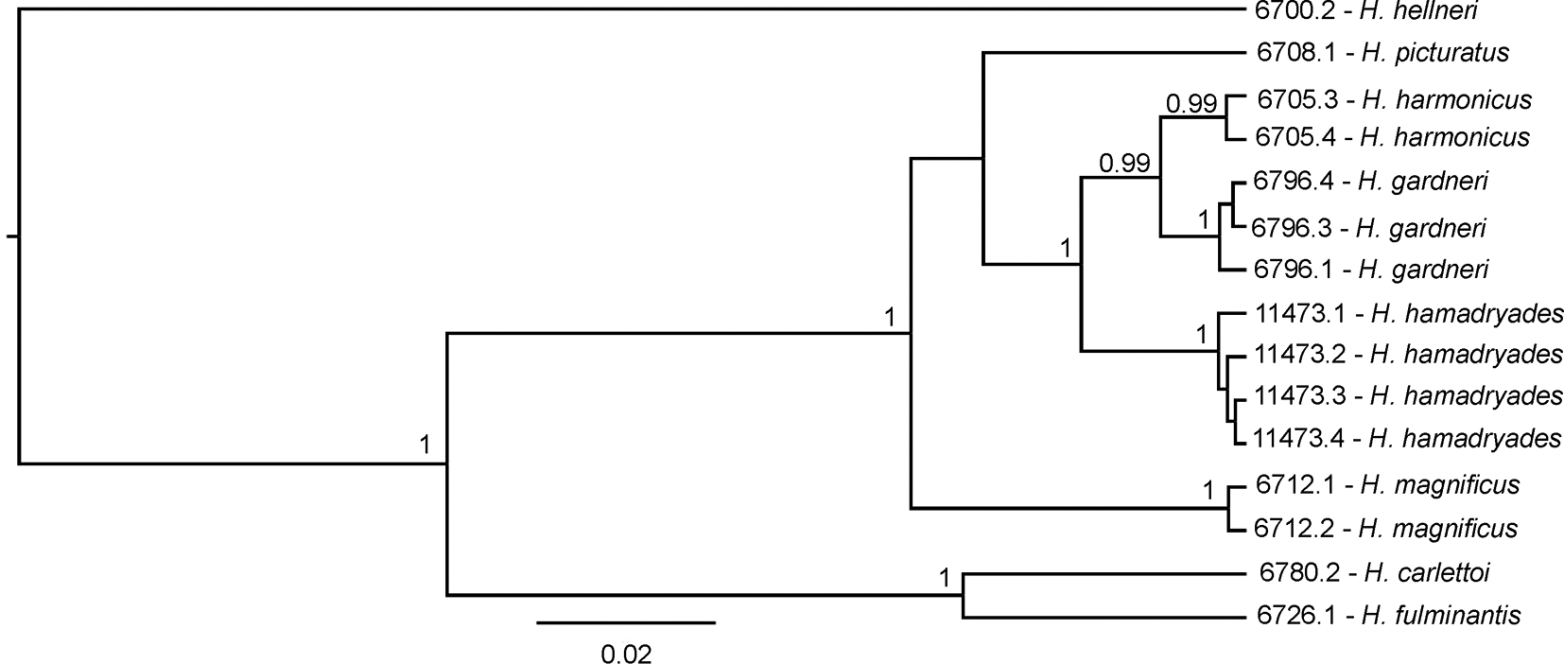
Bayesian phylogeny used to delimit species of the *Hypsolebiasmagnificus* complex inferred by using sequences of the mitochondrial gene cytochrome b, 463 bp. Numbers above nodes are posterior probability values above 95 %; numbers before species names are catalogue numbers for specimens.

BEAST analysis generated a tree with most branches supported by high posterior probability values (0.99–1; Figure 1). All methods of species delimitation yielded identical results, supporting a total of five species within the HMC, including two new species below described. Both new species appear as closely related to *H.harmonicus* in a well-supported clade.

### Taxonomic accounts

#### Hypsolebias gardneri

Taxon classificationAnimaliaCyprinodontiformesRivulidae

Costa sp. n.

http://zoobank.org/1924252C-19C9-42B2-B502-9F2BF8C5867F

Figure 2 , Table 2

##### Material examined.

**Holotype.**UFRJ 11859, male, 36.9 mm SL; Brazil: Bahia state: Malhada municipality: temporary pool near road BR-030, about 8 km NE of the village of Malhada, São Francisco River floodplains, 14°17'39"S, 43°42'32"W, altitude about 440 m above sea level (a.s.l.); W. J. E. M. Costa et al., 31 January 2010. **Paratypes.**UFRJ 6797, 3 males, 29.7–36.1 mm SL, 2 females, 27.9–30.0 mm SL; UFRJ 11860, 2 males, 30.6–33.0 mm SL, 2 females, 26.6–27.9 mm SL (C&S); UFRJ 6796, 3 males, 32.6–36.8 mm SL, 4 females, 26.5 – 29.3 mm SL (DNA); CICCAA02038, 2 males, 32.9–33.6 mm SL; all collected with holotype.

##### Diagnosis.

*Hypsolebiasgardneri* differs from all other species of the *H.magnificus* complex, except *H.harmonicus*, by the following combination of character states relative to the male colour pattern: anterior part of the flank with three dark greenish grey bars (vs. dark greenish grey bars absent in *H.hamadryades*); dorsal fin with transverse blue stripes and one basal row of blue dots (vs. rows of blue dots on the whole fin in *H.picturatus*); anal fin with dots and short vermiculate marks irregularly arranged on the anterior part of the fin (vs. dots on the entire fin in *H.picturatus*, and transverse blue stripes on most fin in *H.magnificus* and *H.hamadryades*); and anterior half of caudal fin with transverse rows of blue dots, posterior half with transverse blue bars (vs. blue bars on most fin in *H.magnificus* and dots on the entire fin in *H.picturatus*). *Hypsolebiasgardneri* is distinguished from *H.harmonicus* by having the caudal fin with 23 or 24 rays, subtruncate and longer in males, measuring 34.5–36.4% SL (vs. with 22 or 22 rays, round, measuring 31.2–33.2% SL), and from *H.hamadryades* by having the dorsal-fin origin just posterior to anal-fin origin in males (vs. anterior) and between the base of 3^rd^ and 5^th^ anal-fin rays in females (vs. between the base of 1^st^ and 3^rd^ anal-fin rays), and the second proximal radial of the dorsal fin between neural spines of the 7^th^ and 8^th^ vertebrae in males (vs. between neural spines of the 5^th^ and 7^th^).

**Figure 2.**
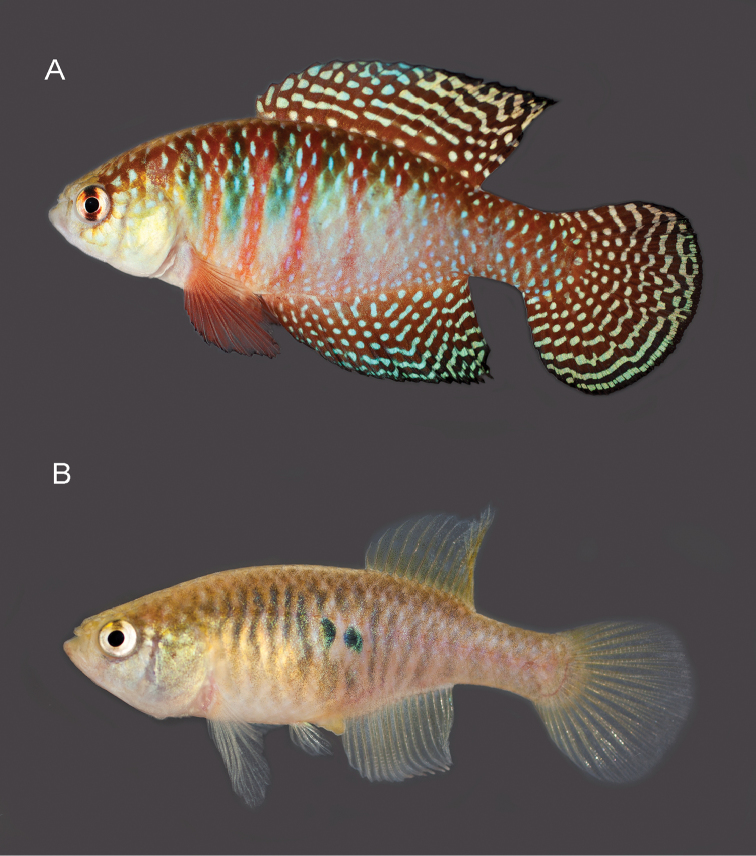
*Hypsolebiasgardneri* sp. n. **A** live holotype, UFRJ 11859, male, 36.9 mm SL**B** live paratype, UFRJ 6797, female, 30.0 mm SL. Photographs by WJEM Costa.

##### Description.

Morphometric data appear in Table 2. Body relatively deep, compressed. Greatest body depth at vertical just anterior to pelvic-fin base. Dorsal and ventral profiles of head and trunk slightly convex, approximately straight on caudal peduncle. Head narrow, sub-triangular in lateral view. Jaws short, teeth numerous, conical, irregularly arranged; outer teeth hypertrophied, inner teeth small and numerous. Vomerine teeth absent. Gill-rakers on first branchial arch 3 + 9, gill-rakers long, straight, without denticles. Urogenital papilla conical in males, pocket-shaped in females, slightly projecting over anterior part of anal fin.

**Table 2.** Morphometric data of *Hypsolebiasgardneri* sp. n.

	Holotype	Paratypes	
	male	males (6)	females (4)
**Standard length (mm)**	**36.9**	**29.7–36.1**	**26.6–30.0**
**Percent of standard length**			
Body depth	36.7	36.4–38.5	33.9–38.4
Caudal peduncle depth	16.4	16.8–17.3	16.0–16.9
Pre-dorsal length	43.1	42.0–47.2	61.1–61.8
Pre-pelvic length	42.8	43.5–45.2	51.9–52.7
Length of dorsal-fin base	45.9	43.4–46.2	24.1–25.9
Length of anal-fin base	43.1	41.1–43.6	23.4–25.4
Caudal-fin length	34.5	34.7–36.4	33.3–36.8
Pectoral-fin length	25.5	26.9–28.8	22.7–26.9
Pelvic-fin length	10.2	10.1–11.7	9.4–11.9
Head length	27.6	27.3–29.8	29.0–31.6
**Percent of head length**			
Head depth	115.6	108.6–121.9	100.1–104.5
Head width	62.2	65.0–71.5	67.3–71.8
Snout length	15.9	13.4–16.3	13.3–14.3
Lower jaw length	20.5	18.8–21.2	15.7–16.8
Eye diameter	30.5	27.9–33.8	30.5–31.6

Dorsal and anal fins relatively short, extremities rounded to slightly pointed in both sexes, without filamentous rays. Caudal fin subtruncate in males, rounded in females. Pectoral fin elliptical, posterior margin reaching between base of 6^th^ and 9^th^ anal-fin ray in males, reaching anus in females. Pelvic fin small, tip reaching between base of 3^rd^ and 5^th^ anal-fin rays in males, reaching base of 1^st^ anal-fin ray in females; pelvic-fin bases medially united. Dorsal-fin origin at vertical between base of 1^st^ and 2^nd^ anal-fin rays in males, between base of 3^rd^ and 5^th^ anal-fin rays in females. Dorsal-fin rays 22–24 in males, 15–17 in females; anal-fin rays 21–22 in males, 17–19 in females; caudal-fin rays 23–24; pectoral-fin rays 12–; pelvic-fin rays 5–6. In males, minute papillate contact organs on inner surface three dorsal-most pectoral-fin rays. Second proximal radial of dorsal fin between neural spines of 7^th^ and 8^th^ vertebrae in males, between neural spines of 11^th^ and 12^th^ vertebrae in females; first proximal radial of anal fin between pleural ribs of 6^th^ and 8^th^ vertebrae in males, between pleural ribs of 8^th^ and 9^th^ vertebrae in females; total vertebrae 26–27.

Scales small, cycloid. Body and head entirely scaled, except anterior ventral surface of head. Body squamation extending over anterior 20% of caudal-fin base and gently extending on middle portion of anal-fin base; no scales on dorsal and pectoral-fin bases. Frontal squamation E-patterned; E-scales overlapping medially; no row of scales anterior to G-scale; supraorbital scales 1–2. Longitudinal series of scales 25–26; transverse series of scales 10; scale rows around caudal peduncle 12. One minute contact organ per scale of ventral portion of flank. Cephalic neuromasts: supraorbital 11–14; parietal 2; anterior rostral 1, posterior rostral 1; infraorbital 2 + 20–24; preorbital 3–4; otic 1–2, post-otic 2–3; supratemporal 1; median opercular 1, ventral opercular 2; pre-opercular 15–17, mandibular 10; lateral mandibular 4, paramandibular 1.

##### Colouration in life.

**Males**. Flank light blue on middle, light pink ventrally, and dark reddish orange dorsally and posteriorly; six to eight light red bars between humeral region and anterior part of caudal peduncle, more conspicuous anteriorly, three anterior-most red bars alternating with three dark greenish grey bars; minute vertically elongated metallic blue spots per scale, on whole flank. Dorsum pale reddish orange, venter white. Head light blue, margin of scales of dorso-posterior region reddish orange to golden. Iris yellow, with dark reddish brown bar through orbit centre. Unpaired fins dark red with bright blue marks, narrower than interspace, including six to nine transverse stripes and one basal row of dots on dorsal fin, stripes often interrupted and substituted by dots on posterior portion of sub-basal portion; dots and short vermiculate marks irregularly arranged on anterior portion of anal fin and transverse stripes on posterior portion; and seven or eight transverse rows of dots on caudal fin, coalesced to form bars on posterior half of fin; each unpaired fin with black line along distal margin. Paired fins red with black margin; minute light blue dots on pelvic fin. **Females.** Flank pale brownish grey, with faint vertically elongated grey spots and short bars along flank and one or two small black spots on flank centre at vertical between anus and anal-fin origin; anterior portion of flank pale golden. Dorsum pale brown, venter white. Head side pale blue with pale golden iridescence on opercle. Iris silver, with dark brownish grey bar through orbit centre. Fins hyaline.

##### Colouration in alcohol.

Males with similar colour pattern as in life, but iridescence is lost and red marks substituted by grey or inconspicuous. Females with similar colour pattern as in life, but iridescence in head is lost.

##### Distribution.

**Figure 3.**
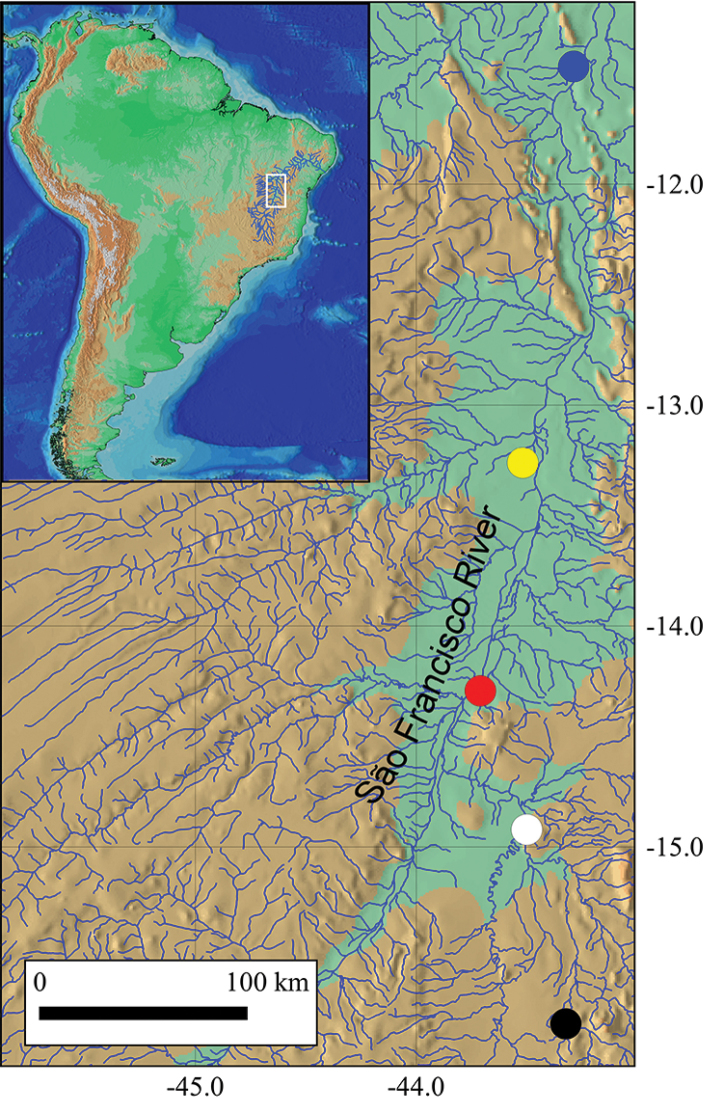
Localities of specimens belonging to species of the *Hypsolebiasmagnificus* complex used in this study: black dot, *H.hamadryades*; white dot, *H.magnificus*; red dot, *H.gardneri*; yellow dot, *H.harmonicus*; blue dot, *H.picturatus*.

*Hypsolebiasgardneri* is known only from the type locality (14°17'39"S, 43°42'32"W, altitude about 500 m a.s.l.; Figure 3), a wide temporary pool, with dense aquatic vegetation in open areas and bushes concentrated on part of the pool bank. Specimens of *Hypsolebiaspterophyllus* Costa, 2012 were common in all parts of the pool, whereas specimens of *H.gardneri* have their distribution restricted to shadow areas, under marginal bushes. The pool was sampled a single time (31 January 2010), when the whole type series was collected. No similar pools were found in the region, thus it is not possible to evaluate its conservation status.

##### Etymology.

The name *gardneri* in honour of Scottish naturalist George Gardner, who was in the Caatinga during his trip to Brazil between 1836 and 1841, making rich natural history collections. His reports on the region, and the numerous plant species and Cretaceous fossil fish collected by him represent important landmarks of our knowledge about Caatinga biodiversity.

#### Hypsolebias hamadryades

Taxon classificationAnimaliaCyprinodontiformesRivulidae

Costa sp. n.

http://zoobank.org/9057BBBC-39AA-4BD9-B84B-005499635FCD

Figure 4 , Table 3

##### Holotype.

UFRJ 6893, male, 26.9 mm SL; Brazil: Minas Gerais state: Janaúba municipality: temporary pool near road MG-401 at the town of Janaúba, floodplains of Gorutuba River, Verde Grande River drainage, São Francisco River basin, 15°48'06"S, 43°19'19"W, altitude about 530 m a.s.l.; W. J. E. M. Costa et al., 17 January 2017.

##### Paratypes.

UFRJ 6895, 4 males, 17.4–26.1 mm SL, 1 female, 21.2 mm SL; UFRJ 6894, 3 males, 22.6 – 26.1 mm SL, 2 females, 15.9–21.0 mm SL (C&S); UFRJ 11473, 4 males, 15.5–17.9 mm SL (DNA); collected with holotype. – UFRJ 6892, 2 females, 21.0–23.7 mm SL; same locality and collectors, 21 April 2017.

##### Diagnosis.

*Hypsolebiashamadryades* is distinguished from all other species of the *H.magnificus* complex by the absence of dark greenish grey bars on the anterior portion of the flank in males (vs. presence) and presence of transverse blue stripes on the unpaired fins in males wider than interspace (vs. transverse series of dots or stripes narrower than interspace). It also differs from all other species of the complex by the following combination of character states relative to the male colour pattern: dorsal fin with transverse blue stripes and one basal row of blue dots (vs. rows of blue dots on the whole fin in *H.picturatus*); anal fin with transverse blue stripes on most portion of the fin (vs. dots and short vermiculate marks arranged on most part of the fin in *H.gardneri*, *H.harmonicus* and *H.picturatus*); and most portion of caudal fin with blue bars (vs. anterior half of caudal fin with transverse rows of blue dots, posterior half with transverse blue bars in *H.gardneri* and *H.harmonicus*, or dots on the entire fin in *H.picturatus*), and the presence of eight to ten light red bars between humeral region and the anterior part of the caudal peduncle (vs. six or seven in *H.magnificus*, *H.harmonicus* and *H.gardneri*). *Hypsolebiashamadryades* is further distinguished from *H.gardneri* and *H.picturatus* by having the dorsal-fin origin anterior to anal-fin origin in males (vs. just posterior anterior) and from *H.harmonicus* by having 23 or 24 caudal-fin rays (vs. 21 or 22).

**Figure 4.**
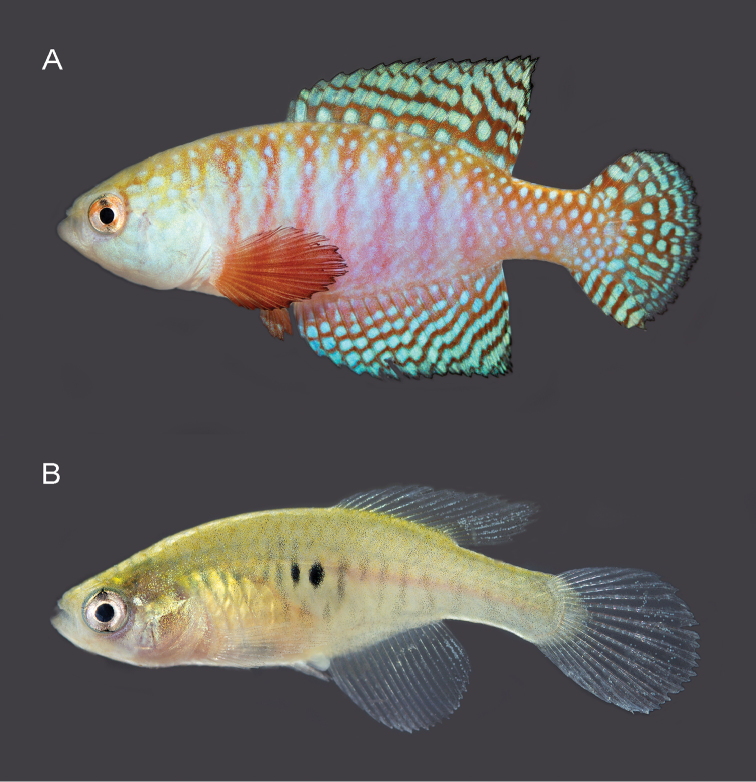
*Hypsolebiashamadryades* sp. n. **A** live holotype, UFRJ 6893, male, 26.9 mm SL (caudal fin damaged and regenerated) **B** live paratype, UFRJ 6895, female, 21.2 mm SL. Photographs by WJEM Costa.

##### Description.

Morphometric data appear in Table 3. Body relatively deep, compressed. Greatest body depth at vertical just anterior to pelvic-fin base. Dorsal and ventral profiles of head and trunk slightly convex, approximately straight on caudal peduncle. Head narrow, sub-triangular in lateral view. Jaws short, teeth numerous, conical, irregularly arranged; outer teeth hypertrophied, inner teeth small and numerous. Vomerine teeth absent. Gill-rakers on first branchial arch 3 + 9, gill-rakers short, straight, without denticles. Urogenital papilla conical in males, pocket-shaped in females, slightly projecting over anterior part of anal fin.

**Table 3.** Morphometric data of *Hypsolebiashamadryades* sp. n. Caudal fin of holotype damaged and regenerated, thus not measured.

	Holotype	Paratypes	
	male	males (3)	females (3)
**Standard length (mm)**	**26.9**	**24.4–26.1**	**21.0–23.7**
**Percent of standard length**			
Body depth	36.0	33.9–35.0	33.9–34.6
Caudal peduncle depth	15.3	14.2–14.8	12.9–14.9
Pre-dorsal length	45.6	44.7–45.1	61.1–61.5
Pre-pelvic length	44.3	42.6–44.4	52.8–53.6
Length of dorsal-fin base	44.7	40.1–45.5	23.7–25.2
Length of anal-fin base	41.1	36.7–41.7	22.6–23.9
Caudal-fin length	-	33.0–33.9	32.7–34.9
Pectoral-fin length	25.0	25.5–28.0	23.9–26.5
Pelvic-fin length	9.6	8.2–10.6	8.5–11.9
Head length	29.1	28.1–30.4	28.3–31.1
**Percent of head length**			
Head depth	101.3	99.7–102.6	97.1–100.6
Head width	63.6	59.8–61.5	66.5–70.5
Snout length	14.2	13.7–15.8	11.9–13.9
Lower jaw length	19.4	16.5–18.8	14.1–16.5
Eye diameter	31.1	31.6–33.9	34.2–36.1

Dorsal and anal fins relatively short, extremities rounded to slightly pointed in both sexes, without filamentous rays. Caudal fin rounded. Pectoral fin elliptical, posterior margin reaching base of 7^th^ anal-fin ray in males, reaching anus in females. Pelvic fin small, tip reaching between base of 2^nd^ and 3^rd^ anal-fin rays in males, reaching urogenital papilla in females; pelvic-fin bases medially united. Dorsal-fin origin anterior to anal-fin origin in males, anal-fin origin at vertical between base of 1^st^ and 3^rd^ dorsal-fin rays; dorsal-fin origin posterior to anal-fin origin in females, dorsal-fin origin at vertical between base of 1^st^ and 3^rd^ anal-fin rays in females. Dorsal-fin rays 22–26 in males, 15–17 in females; anal-fin rays 20–23 in males, 18–19 in females; caudal-fin rays 23–24; pectoral-fin rays 12–13; pelvic-fin rays 5–6. In males, minute papillate contact organs on inner surface of dorsal-most pectoral-fin ray. Second proximal radial of dorsal fin between neural spines of 5^th^ and 7^th^ vertebrae in males, between neural spines of 10^th^ and 12^th^ vertebrae in females; first proximal radial of anal fin between pleural ribs of 7^th^ and 9^th^ vertebrae in males, between pleural ribs of 8^th^ and 10^th^ vertebrae in females; total vertebrae 27–29.

Scales small, cycloid. Body and head entirely scaled, except anterior ventral surface of head. Body squamation extending over anterior 25% of caudal-fin base; no scales on dorsal, anal and pectoral-fin bases. Frontal squamation E-patterned; E-scales overlapping medially; no row of scales anterior to H-scale; one supraorbital scale. Longitudinal series of scales 26; transverse series of scales 11; scale rows around caudal peduncle 12. One minute contact organ per scale of anteroventral portion of flank. Cephalic neuromasts: supraorbital 14–16; parietal 2; anterior rostral 1, posterior rostral 1; infraorbital 2 + 18–20; preorbital 2; otic 2–3, post-otic 2; supratemporal 1; median opercular 1, ventral opercular 1; pre-opercular 12–15, mandibular 10; lateral mandibular 5, paramandibular 1.

##### Colouration in life.

**Males**. Flank light blue on middle, light pink ventrally, and pale reddish orange dorsally and posteriorly; eight to ten light red bars between humeral region and anterior part of caudal peduncle, more conspicuous anteriorly; minute vertically elongated metallic blue spots per scale, on whole flank. Dorsum pale reddish orange, venter white. Head light blue, margin of scales of dorso-posterior region reddish orange. Iris yellow, with dark reddish brown bar through orbit centre. Unpaired fins red with bright blue transverse stripes, sometimes interrupted, including four or five on dorsal and anal fins, and six or seven on caudal fin; stripes broader than interspace; each unpaired fin with one row of small bright blue spots along basal portion and black line along distal margin. Paired fins red with black margin; faint blue dots on pelvic fin. **Females.** Flank light grey, with faint vertically elongated grey spots and one or two small black spots on flank centre, at vertical between pelvic-fin base and urogenital papilla; scale border pale yellow on dorsal portion of flank and head. Dorsum light grey, venter white. Head side pale grey with pale golden iridescence on opercle. Iris silver, with dark grey bar through orbit centre. Fins hyaline.

##### Colouration in alcohol.

Males with similar colour pattern as in life, but iridescence is lost and red marks substituted by grey or inconspicuous. Females with similar colour pattern as in life, but iridescence in head is lost.

##### Distribution and conservation.

*Hypsolebiashamadryades* is only known from a pool in the floodplains of the Gorutuba River, within the town of Janaúba, Minas Gerais, Brazil (15°48'06"S, 43°19'19"W, altitude about 530 m a.s.l.; Figure 3). This area has been studied since January 2002 (Costa, 2006), but *H.hamadryades* was first collected only in 2017. Previous field studies revealed two endemic seasonal killifishes, *H.janaubensis* (Costa, 2006) and *Cynolebiasgorotuba* Costa, 2017, as well as an intense process of urbanization which result in the complete extirpation of all temporary pools studied between 2002 and 2010 (Costa 2017). The type locality pool of *H.hamadryades* was only found in January 2017, since it was hidden by a dense Caatinga forest. The pool occupied an area of about 100 m^2^ and was about 1 m deep. The whole pool was densely populated by adult specimens of *H.janaubensis*, whereas individuals of *H.hamadryades*, mostly juvenile specimens below 20 mm SL including, were found only in a small part of the pool containing shaded zones, near the pool margins, where bushes were concentrated. The largest males exhibited damaged caudal fins, indicating possible territorial disputes as commonly occurring in other seasonal killifishes. A new collecting trip was made in April 2017, when physical conditions of the pool were nearly identical to the first collection, except that the pool was shallower (about 0.5 m at deepest places). At that time, however, only two females and no males of *H.hamadryades* were found. According to local people, pools in the region did not dry between January and the period of the second collection.

##### Etymology.

The name *hamadryades* is an allusion to the occurrence of the new species in the forested part of a Caatinga temporary pool. This name was used by the Bavarian naturalist Karl Friedrich Philipp von Martius for the Caatinga in his classification of vegetation formations of Brazil, in which he used names of Greek mythological beings to name each Brazilian phytogeographical province. The name is opportune by referring to *hamadryades*, a particular kind of Greek nymph entity that is believed to be associated to trees, vanishing when trees die. Similarly, field studies have shown that populations of species of the *H.magnificus* group became extinct after marginal deforestation (see discussion below).

## Discussion

**Figure 5.**
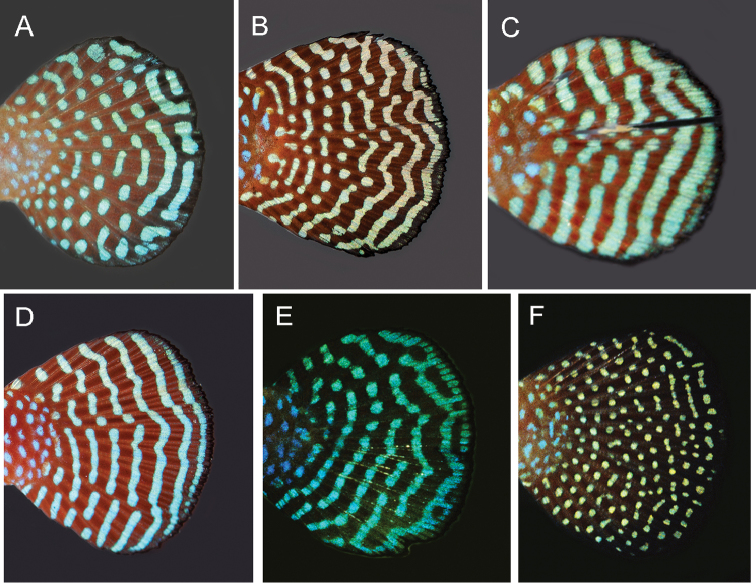
Caudal fin of live males of the *Hypsolebiasmagnificus* species complex. **A***H.harmonicus*, holotype, UFRJ 6696, 29.4 mm SL**B***H.gardneri* sp. n., paratype, UFRJ 6797, 34.4 mm SL**C***H.hamadryades* Costa sp. n., paratype, UFRJ 6895, 24.8 mm SL**D***H.magnificus*, specimen from Gado Bravo, UFRJ 4959, 31.2 mm SL**E***H.magnificus*, topotype not preserved, about 25 mm SL**F***H.picturatus*. Paratype, UFRJ 5053, 38.6 mm SL. Photographs by WJEM Costa.

The two new species here described, *H.gardneri* and *H.hamadryades*, are respectively most similar in colour pattern to *H.harmonicus* and *H.magnificus*, what is mostly evident in the male caudal fin (Figure 5). In both *H.gardneri* and *H.harmonicus*, the caudal fin of males is blue-dotted on its proximal portion and has light blue bars on the distal one (Figs 2A, 5A, B), and in *H.hamadryades* and *H.magnificus* there are light blue bars scattered over the whole fin (Figure 5C–E), thus contrasting with the entire blue-dotted fin in *H.picturatus* (Figure 5F). Whereas the molecular analysis corroborates *H.gardneri* and *H.harmonicus* as sister species (Figure 1), *H.hamadryades* is not supported as closer to *H.magnificus*, but to *H.gardneri* and *H.harmonicus*. *Hypsolebiasgardneri* and *H.harmonicus* share a similar colour pattern on the caudal fin, unique among congeners, but the former species has a proportionally larger fin (34.5–36.4% SL) that is subtruncate (Figs 2A, 5B) and bears more rays (23–24), whereas the fin is conspicuously smaller (31.2–33.2% SL) and rounded (Figure 5A), besides having fewer rays (21–22). In contrast to all other species of HMC, in *H.hamadryades* the bars on the caudal fin are wider than the dark red interspace (Figure 5C).

Little is presently known about the maximum length that seasonal killifishes endemic to the Caatinga may reach. According to field data, species of the genus *Cynolebias* Steindachner, 1876 may reach about 135 mm SL (Costa 2001), whereas species of the *H.antenori* group reach about 75 mm SL (Costa 2007), but species of the *H.magnificus* group do not surpass about 45 mm SL (Costa 2007). Among species of HMC, *H.gardneri*, *H.magnificus*, and *H.picturatus* may reach between 42–45 mm SL, but *H.hamadryades* and *H.harmonicus* seem to be smaller. *Hypsolebiashamadryades* was first collected in January 2017, when specimens found were always small, males reaching a maximum size of 27 mm SL and females 21 mm SL. A new collection was made three months after, just before the long dry period. However, only females were found, which exhibited approximately the same size as in January, although local people reporting that pools had not dried between the two trips. The only other seasonal fish found in sympatry, *H.janaubensis*, measured about 50 mm SL, the largest size recorded for this species, suggesting that seasonal killifishes of the pool were at their maximum size. These data suggest that *H.hamadryades* is a miniature species, probably constituting the smallest species of the genus.

Although our phylogeny was based in a short fragment of a single mitochondrial gene and relatively low number of individuals, the concordance between molecularly delimited (i.e., exclusive lineages supported by high Bayesian posterior probabilities) and morphologically diagnosable (i.e., exhibiting unique combination of morphological character states) highly supports recognition of five distinct species. In addition, the naturally fragmented distribution pattern of recognised species due to their ecologically specialised nature in uniquely inhabiting pools under dense concentration of shrubs and trees, which is not a common habitat in the Caatinga, also reinforces hypotheses of genetic isolation among these species.

Continuous field studies in the Caatinga have revealed a high diversity of seasonal killifish species, but they have also reported rapid environmental decline caused by deforestation, new roads and draining projects for agricultural proposals, extirpating natural habitats many killifish populations (Costa 2002, 2017; Costa et al. 2012). As a consequence, several of these species have appeared in lists of endangered species (e.g., http://www.icmbio.gov.br/portal/especies-ameacadas-destaque). However, our field studies have clearly shown that species of the *H.magnificus* group are much more susceptible to extinction than most other seasonal killifishes of the region. Besides being rarer, occurring only in areas where terrestrial marginal vegetation is taller, they are highly sensitive to deforestation, tending to disappear just after original vegetation is removed, whereas species of the *H.antenori* group and *Cynolebias* may survive in areas highly exposed to sunlight (pers. observ. 2002–2018). This study therefore indicates that HMC comprises cryptic species apparently endemic to small areas and extremely vulnerable to environmental changes, deserving high concern.

## Supplementary Material

XML Treatment for Hypsolebias gardneri

XML Treatment for Hypsolebias hamadryades
